# Unleashing the Diagnostic, Prognostic and Therapeutic Potential of the Neuronostatin/GPR107 System in Prostate Cancer

**DOI:** 10.3390/jcm9061703

**Published:** 2020-06-02

**Authors:** Prudencio Sáez-Martínez, Juan M. Jiménez-Vacas, Antonio J. León-González, Vicente Herrero-Aguayo, Antonio J. Montero Hidalgo, Enrique Gómez-Gómez, Rafael Sánchez-Sánchez, María J. Requena-Tapia, Justo P. Castaño, Manuel D. Gahete, Raúl M. Luque

**Affiliations:** 1 Maimonides Institute for Biomedical Research of Córdoba (IMIBIC), 14004 Cordoba, Spain; prudensama95@gmail.com (P.S.-M.); b12jivaj@uco.es (J.M.J.-V.); bc2legoa@uco.es (A.J.L.-G.); b22heagv@uco.es (V.H.-A.); b42mohia@uco.es (A.J.M.H.); enriquegomezgomez@yahoo.es (E.G.-G.); patologiahrs@gmail.com (R.S.-S.); josefa.requena.sspa@juntadeandalucia.es (M.J.R.-T.); bc1cafuj@uco.es (J.P.C.); bc2gaorm@uco.es (M.D.G.); 2 Department of Cell Biology, Physiology, and Immunology, University of Córdoba, 14071 Cordoba, Spain; 3 Hospital Universitario Reina Sofía (HURS), 14004 Cordoba, Spain; 4 Centro de Investigación Biomédica en Red de Fisiopatología de la Obesidad y Nutrición, (CIBERobn), 14004 Cordoba, Spain; 5 Urology Service, HURS/IMIBIC, 14004 Cordoba, Spain; 6 Anatomical Pathology Service, HURS, 14004 Cordoba, Spain

**Keywords:** prostate cancer, castration resistant prostate cancer, neuronostatin, G protein-coupled receptor GPR107, diagnostic/prognostic biomarker, therapeutic target, somatostatin-system, splicing

## Abstract

Certain components of the somatostatin-system play relevant roles in Prostate Cancer (PCa), whose most aggressive phenotype (Castration-Resistant-PCa (CRPC)) remains lethal nowadays. However, neuronostatin and the G protein-coupled receptor 107 (GPR107), two novel members of the somatostatin-system, have not been explored yet in PCa. Consequently, we investigated the pathophysiological role of NST/GPR107-system in PCa. GPR107 expression was analyzed in well-characterized PCa patient′s cohorts, and functional/mechanistic assays were performed in response to GPR107-silencing and NST-treatment in PCa cells (androgen-dependent (AD: LNCaP) and androgen-independent (AI: 22Rv1/PC-3), which are cell models of hormone-sensitive and CRPC, respectively), and normal prostate cells (RWPE-1 cell-line). GPR107 was overexpressed in PCa and associated with key clinical parameters (e.g., advance stage of PCa, presence of vascular invasion and metastasis). Furthermore, GPR107-silencing inhibited proliferation/migration rates in AI-PCa-cells and altered key genes and oncogenic signaling-pathways involved in PCa aggressiveness (i.e., KI67/CDKN2D/MMP9/PRPF40A, SST_5_TMD4/AR-v7/In1-ghrelin/EZH2 splicing-variants and AKT-signaling). Interestingly, NST treatment inhibited proliferation/migration only in AI-PCa cells and evoked an identical molecular response than GPR107-silencing. Finally, NST decreased GPR107 expression exclusively in AI-PCa-cells, suggesting that part of the specific antitumor effects of NST could be mediated through a GPR107-downregulation. Altogether, NST/GPR107-system could represent a valuable diagnostic and prognostic tool and a promising novel therapeutic target for PCa and CRPC.

## 1. Introduction

Prostate cancer (PCa) is one of the most diagnosed tumor pathologies among men worldwide and represents one of the leading causes of cancer-related death among the male population in developed countries [1]. One of the main characteristics of PCa is the strong influence of the endocrine-metabolic environment, which contributes to the high heterogeneity of this pathology and hinders the development of new, useful, diagnostic and/or therapeutic strategies [2]. In fact, the current available pharmacological approaches used in clinical practice to combat this tumor pathology consist in the use of drugs (e.g., Abiraterone, Enzalutamide, etc.) that avoid the steroid-hormones synthesis and/or disrupts the androgen signaling pathway in PCa cells [3,4]. However, although the development of these drugs has significantly improved the overall survival, some PCa patients acquire resistance to these compounds and develop the most aggressive phenotype, named Castration-Resistant PCa (CRPC), which remains lethal nowadays [3,4,5]. For this reason, new therapeutic targets in order to tackle PCa and CRPC are urgently required.

In this sense, certain components of the somatostatin system, especially somatostatin-receptors (SST_1-5_, encoded by the somatostatin receptor 1-5 genes (SSTR1-5)), are expressed in both normal and tumor prostate tissues, where they may play a relevant role in the development and progression of this disease [6,7,8,9]. Specifically, some components of this system (e.g., SST_1_ and SST_2_) can contribute to reduce different tumor parameters, including cell proliferation and migration, whereas other components [i.e., the truncated splicing variant of SST_5_ with four transmembrane domains (SST_5_TMD4 variant)] promote aggressiveness features of PCa [10,11,12]. Synthetic somatostatin analogs (i.e., octreotide and/or pasireotide) have been used as valuable tools to treat different tumor pathologies, including pituitary and neuroendocrine tumors [13]. However, attempts to apply somatostatin analogs in PCa have rendered controversial results, since the limited studies reported so far did not show improvement in overall survival [14]. The mechanistic reasons of those clinical failures are still unknown, but it has been suggested that one of the causes might be the overexpression of the spliced variant SST_5_TMD4 in PCa cells, which hampers the normal response to somatostatin-analogs in PCa-cells [11].

Interestingly, the complexity and versatility of the somatostatin system has been lately increased by the discovery of a new peptide contained in the preprosomatostatin precursor polypeptide encoded by the somatostatin gene, which shares no amino-acid homology to somatostatin, named neuronostatin (NST) [15]. NTS seems to bind to the G protein-coupled receptor 107 (GPR107) to exert its actions [16]. Specifically, it has been reported that NST and/or GPR107 are expressed in different tissues (e.g., brain, pancreas, gut, etc.) wherein they exert important pathophysiological functions, being some of these actions similar, but others also unique, to those exerted by other members of the somatostatin/SSTRs-system [17,18,19]. Despite the tight genetic and putative functional link between NST and somatostatin, the presence and/or functional role of the NST/GPR107-system has hitherto not been explored on PCa. Accordingly, the current study sought to explore for the first time the functional actions and therapeutic potential of the NST/GPR107-system in PCa cells by investigating the direct effects of NST treatment on normal and tumor (PCa and CRPC) cells and the pathophysiological role of endogenous GPR107 in this severe disease.

## 2. Experimental Section

### 2.1. Patients and Samples

This study was approved by the Hospital Ethic Committee (approval number: 2461) and conducted in accordance to the principles of the Declaration of Helsinki. Written informed consent was obtained from all patients. Two different cohorts of prostate samples obtained through the Andalusian Biobank (Cordoba Node) were included:

Cohort 1: formalin-fixed, paraffin-embedded (FFPE) PCa tissues (*n* = 84) and their adjacent non-tumor region (N-TAR; used as control tissues; *n* = 84), which were obtained from radical prostatectomies from patients who were diagnosed with localized PCa, without metastasis and with Gleason Score (GS) 6–8 (Table 1).

Cohort 2: fresh PCa samples (*n* = 67) that were obtained by core needle biopsies from patients with high suspect of presenting palpable significant PCa, which was further confirmed histologically by a specialized pathologist. This cohort includes more aggressive PCa, presenting metastasis in some cases (metastatic hormone-sensitive PCa or mHSPC) and with GS 7–10 (Table 2).

Computed tomography scan and bone scan were performed in these patients to determine the presence of metastasis. Available clinical parameters of tumor aggressiveness were collected from each patient, such as presence of metastasis, Gleason score (analyzed by specialist uro-pathologists following the 2005, 2010 and 2014 International Society of Urological Pathology (ISUP) criteria, based on the sample collection date [20,21,22]) and prostatic specific antigen (PSA) levels (cohort 1 (Table 1) and cohort 2 (Table 2)). In addition, expression and clinical data of interest for this study were downloaded from different available in silico cohorts using cBioPortal (Grasso/Varambally cohorts) [23,24,25] or CANCERTOOL (Lapointe/Taylor/Tomlins) [26,27,28,29]. Specifically, Grasso cohort includes 35 metastatic Castration Resistant Prostate Cancer (mCRPC), 59 localized prostate carcinomas and 28 benign prostate tissue specimens; Varambally cohort includes 6 mCRPC, 7 primary prostate carcinomas and 6 normal prostate samples; Lapointe cohort includes 9 mHSPC, 62 localized prostate carcinomas and 41 matched normal prostate tissues; Taylor cohort includes 19 mHSPC, 131 localized prostate carcinomas and 29 paired normal adjacent prostate tissue specimens and Tomlins cohort includes 19 mHSPC, 49 localized prostate carcinomas and 23 normal prostate glands.

### 2.2. Cell Cultures and Reagents

The androgen-dependent metastatic PCa LNCaP cell line, the androgen-independent 22Rv1 and PC-3 (non-metastatic and metastatic, respectively) PCa cell lines and the normal-like prostate cell line RWPE-1 were obtained from American Type Culture Collection (ATCC, Manassas, VA, USA) and maintained according to manufacturer instructions as previously described [10,11,30]. These cell lines were validated by analysis of short tandem repeats sequences (STRs) using GenePrint 10 System (Promega, Barcelona, Spain) and checked for mycoplasma contamination by polymerase chain reaction (PCR) as previously reported [11]. For functional assays, selected cell lines were used as indicated. For mechanistic assays, 22Rv1 and PC-3 were used as representative models of androgen-independence with and without AR-v7 expression, respectively. Human amidated NST-19(Ala-Pro-Ser-Asp-Pro-Arg-Leu-Arg-Gln-Phe-Leu-Gln-Lys-Ser-Leu-Ala-Ala-Ala-Ala-NH_2_) was purchased from Phoenix Pharmaceuticals (Burlingame, CA, USA), resuspended in water and used at 10^−7^ M based on previous reports [19].

### 2.3. Transfection with Specific siRNA

For silencing assays, 22Rv1 and PC-3 cell lines were used. Specifically, 200,000 cells were seeded in 6-well plates and grown until 70% confluence was reached. Then, cells were transfected with specific small interferent RNA (siRNA) against GPR107 (Catalog # AM16708; Thermo Fisher Scientific, Madrid, Spain) at 15 nM or scramble control (Catalog # 4390843, Thermo Fisher Scientific, Madrid, Spain) using Lipofectamine-RNAiMAX (Catalog # 13778-150, Thermo Fisher Scientific, Madrid, Spain) following the manufacturer’s instructions. After 48 h, cells were collected for validation (quantitative-PCR (qPCR) and western blot) and seeded for proliferation and/or migration assays.

### 2.4. Measurements of Cell Proliferation and Migration Rates

Both cell proliferation and migration were measured as previously reported [11,31]. Briefly, cell proliferation was assessed by Resazurin Reagent (# CA035; Canvax Biotech, Córdoba, Spain) following the manufacturer’s instructions. Cells were seeded in 96-well plates at a density of 3000–5000 cells/well and serum-starved for 24 h, then cell proliferation was evaluated using FlexStation III system (Molecular Devices, Sunnyvale, CA, USA) until 72 h in response to NST-19 treatment and/or GPR107-silencing. All experiments were performed at least with three independent cell preparations.

Cell migration was evaluated by wound-healing assay in RWPE-1 and PC-3 cells due to the inability of LNCaP and 22Rv1 to migrate. Images of the wound were taken at 0 and 24 h, and wound healing was calculated as the area of a rectangle centered in the picture 24 h after the wound was made vs. the area of the rectangle just after doing the wound. Results were expressed as percentage referred to control. All experiments were performed at least with three independent cell preparations.

### 2.5. RNA Isolation, Reverse Transcription and Quantitative Real-Time PCR (RT-qPCR)

Total Ribonucleic acid (RNA) from FFPE samples was isolated and treated with Deoxyribonuclease (DNase) using the Maxwell 16 LEV RNA FFPE Kit (Promega, Madison, WI, USA) according to manufacturer instructions in the Maxwell MDx 16 Instrument (Promega, Madison, WI, USA). Additionally, total RNA was extracted from fresh samples using the AllPrep DNA)/RNA/Protein Mini Kit (Qiagen, Madrid, Spain) and from prostate cell lines using TRIzol Reagent (Thermo Fisher Scientific, Madrid, Spain), followed, in both cases, by DNase treatment using Ribonuclease (RNase)-Free DNase Kit (Qiagen, Hilden, Germany). Total RNA concentration and purity was assessed using Nanodrop One Spectrophotometer (Thermo Fisher Scientific, Madrid, Spain). Total RNA was retrotranscribed using random hexamer primers and the cDNA First Strand Synthesis Kit (Thermo Fisher Scientific, Madrid, Spain).

Details regarding the development, validation, and application of the RT-qPCR to measure expression levels of the transcripts of interest have been previously reported by our laboratory [32,33,34,35]. Specific and validated primers set used to measure the expression levels of genes of interest in this study (absolute mRNA copy number/50 ng of sample) are described in Table S1. To control for variations in the amount of RNA used and the efficiency of the retro-transcription, messenger RNA (mRNA) copy numbers of the different transcripts analyzed were adjusted by a normalization factor, which was calculated with by the expression levels of Actin Beta (*ACTB)* and Glyceraldehyde-3-Phosphate Dehydrogenase (*GAPDH)* using GeNorm 3.3 (CMMG, Ghent, Belgium) [36] or by the expression levels of *ACTB* (the most appropriated housekeeping gene), wherein *ACTB* and *GADPH* mRNA levels did not significantly vary among the different experimental conditions.

### 2.6. Western-Blotting

Prostate cell lines were processed to analyze protein levels by western-blot after 5, 10 and 15 min of NST-19 exposure and after 48 h of GPR107 siRNA transfection. These processes have been previously described by our group [11,30,34]. Briefly, 150,000 cells were seeded in 12-well plates, and after the experimental procedure describe above, proteins were extracted using pre-warmed Sodium Dodecyl Sulfate-Dithiothreitol (SDS-DTT) buffer (62.5 mM Tris-HCl, 2% SDS, 20% glycerol, 100 mM DTT, and 0.005% bromophenol blue). Then, proteins were sonicated for 10 s and boiled for 5 min at 95 °C. Proteins were separated by SDS-PAGE and transferred to nitrocellulose membranes (Millipore, Billerica, MA, USA). Membranes were blocked with 5% non-fat dry milk in Tris-buffered saline/0.05% Tween-20 and incubated overnight with the specific primary antibodies for GPR107 (orb161193; byorbyt, Cambridge, United Kingdom), Tubulin Beta (TUBB; # 2128S; Cell-Signaling, Barcelona, Spain), phospho-AKT (p-AKT; #4060S; Cell-Signaling, Barcelona, Spain) and total-AKT (#9272S; Cell-Signaling, Barcelona, Spain). Secondary horseradish peroxidase (HRP)-conjugated goat anti-rabbit Immunoglobulin G (IgG) (# 7074S; Cell-Signaling, Barcelona, Spain) were used. Proteins were detected using an enhanced chemiluminescence detection system (GEHealthcare, Madrid, Spain) with dyed molecular weight markers (Bio-Rad, Madrid, Spain). A densitometry analysis of the bands obtained was carried out with ImageJ 2 software (Madison, Wisconsin, USA) [37], using total protein levels of TUBB (for GPR107) or AKT (for phospho-AKT) as normalizing factors. All experiments were performed at least with three independent cell preparations.

### 2.7. GPR107 Immunohistochemistry (IHC) Analysis

GPR107 Immunohistochemistry (IHC) analysis was performed on FFPE samples from cohort 1 (*n* = 16; randomly selected samples) and cohort 2 (*n* = 4; metastatic patients) using standard procedures [11,38,39]. In both cases, the staining of the tumor tissue was compared to non-tumor adjacent regions. Briefly, deparaffinized sections were incubated overnight (4 °C) with the primary antibody against GPR107 (# 161193; Biorbyt, Cambridge, United Kingdom) at 1:200 dilution, followed by incubation with an anti-rabbit horseradish peroxidase-conjugated secondary antibody (# 7074; Cell-Signaling, Barcelona, Spain). Finally, sections were developed with 3.39-diaminobenzidine (Envision system 2-Kit Solution DAB, Thermo Fisher Scientific, Madrid, Spain) and contrasted with haematoxylin. Two independent pathologists performed histopathologic analysis of the tumors following a blinded protocol. In the analysis, 1, 2, 3 indicate low, moderate and high staining intensities.

### 2.8. Statistical Analysis

Statistical differences between two conditions were calculated by unpaired parametric t-test or nonparametric Mann Whitney U test, according to normality, assessed by Kolmogorov–Smirnov test. For differences among three conditions, One-Way ANOVA analysis was performed. Spearman’s or Pearson’s bivariate correlations were performed for quantitative variables according to normality. All the experiments were performed in at least 3 experiments (*n* ≥ 3) and with at least 2 technical replicates. The receiver operating characteristic (ROC) curve was performed for evaluation of the accuracy of GPR107 as a discriminator marker between metastatic vs. non-metastatic patients. Statistical significance was considered when *p* < 0.05. A trend for significance was indicated when *p* values ranged between > 0.05 and < 0.1. All the analyses were assessed using GraphPad Prism 7 (GraphPad 7 Software, La Jolla, CA, USA).

## 3. Results

### 3.1. GPR107 is Overexpressed in PCa and Associated with Aggressive Features

**Figure 1:**
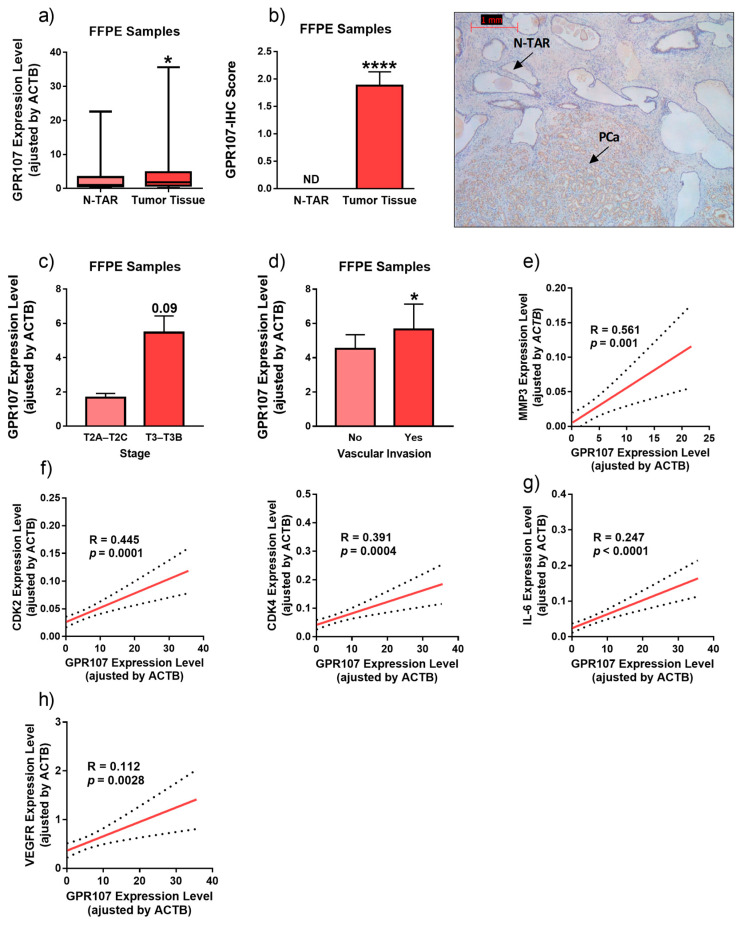
Expression levels of G protein-coupled receptor 107 (GPR107) in human prostate cancer samples. (**a**) Comparison of GPR107 expression levels between formalin-fixed paraffin embedded (FFPE) samples from Prostate Cancer (PCa) tissues and non-tumor adjacent regions (N-TAR) (*n* = 84). (**b**) Comparation of GPR107 protein levels by Immunohistochemistry (IHC) between a representative set of PCa samples (*n* = 16) and its N-TAR (*n* = 16). A representative image is also included. (**c**,**d**) Association between GPR107 expression levels and advance stage of PCa (**c**) and vascular invasion (**d**). (**e**–**h**) Correlation of GPR107 expression levels and Matrix Metallopeptidase 3 (MMP3; (**e**), Cyclin Dependent Kinase 2 and 4 (CDK2 and CDK4, respectively; (**f**), Interleukin 6 Receptor (IL6-R; (**g**) and Vascular endothelial growth factor Receptor (VEGFR; (**h**) expression levels in the same cohort of FFPE samples. Messenger RNA (mRNA) levels were determined by quantitative Polymerase Chain Reaction (qPCR) and adjusted by Actin Beta (ACTB) expression levels. Asterisks (* *p* < 0.05; **** *p* < 0.0001) indicate statistically significant differences between groups. ND: Non-detected.

Analysis of *GPR107* mRNA expression in FFPE-prostate pieces from patients diagnosed with localized PCa (*n* = 84; Gleason score 6–8; Table 1) revealed that GPR107 expression was significantly higher in tumor vs. non-tumor adjacent regions (N-TAR; Figure 1a). GPR107 IHC analysis was performed on 16 FFPE pieces (Figure 1b), which revealed that GPR107 staining was negligible in benign prostate gland epithelium (N-TAR; Figure 1b), while it was always more, and highly, intense in the cancerous prostate glands (N-TAR vs. PCa/tumor-tissue; Figure 1b).

Interestingly, GPR107 expression was associated with key clinical and molecular features of aggressiveness. Specifically, we found a positive association between GPR107 expression and an advance stage of PCa (Tumor Stage 2A-2C vs. Tumor Stage 3-3B (T2A–T2C vs. T3–T3B; Figure 1c)) and with the presence of vascular invasion (Figure 1d), while no association was found with GS. Moreover, GPR107 expression was positively correlated with the expression of key genes related to migration (Matrix Metallopeptidase 3 (MMP3); Figure 1e), cell-cycle control (Cyclin Dependent Kinase 2 and 4 (CDK2 and CDK4, respectively); Figure 1f), inflammatory state (Interleukin 6 Receptor (IL6R); Figure 1g) and angiogenesis process (Vascular endothelial growth factor Receptor (VEGFR); Figure 1h).

### 3.2. GPR107 is Overexpressed in Patients with Metastasis

**Figure 2:**
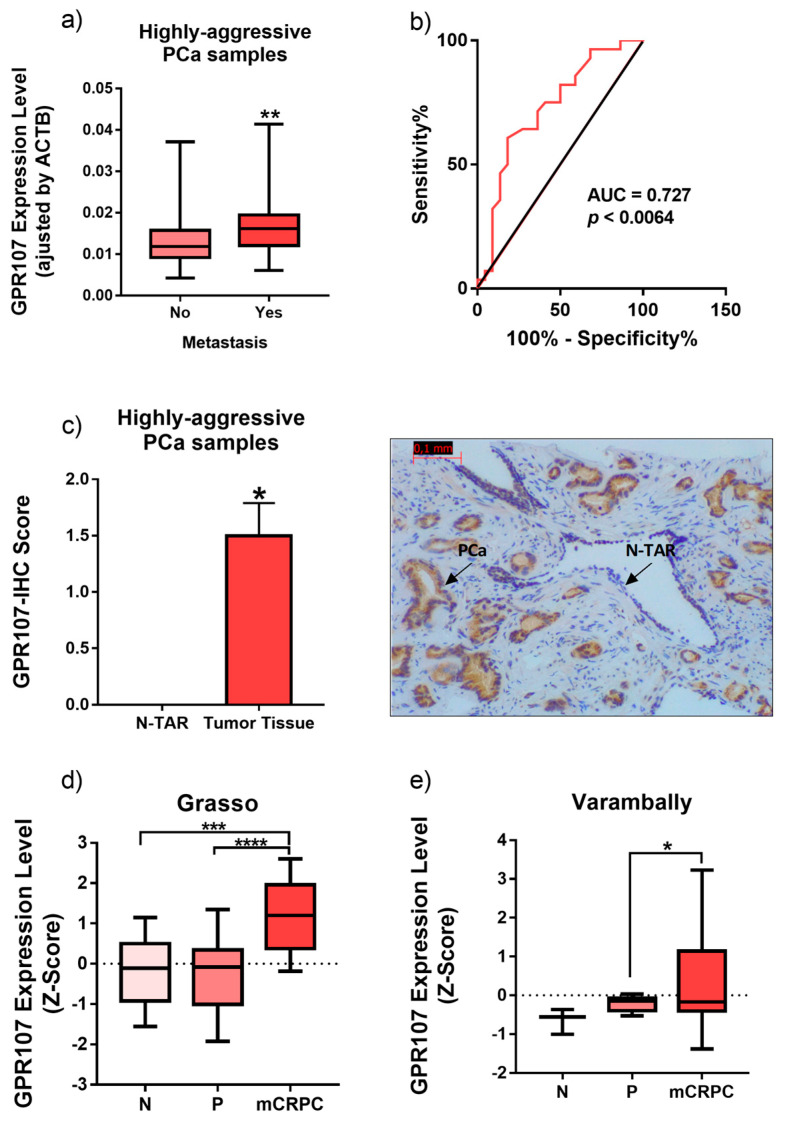
Expression levels of G protein-coupled receptor 107 (GPR107) in primary prostate cancer samples from patients with metastasis. (**a**) Association between GPR107 expression levels and the presence of metastasis in a cohort of fresh samples from patients with Prostate Cancer (PCa) *n* = 67). (**b**) Operating characteristic (ROC) curve analysis to determine the accuracy of GPR107 to discriminate between metastatic vs. non-metastatic patients’ tumor. (**c**) Comparison of GPR107 protein level by Immunohistochemistry (IHC) between primary PCa samples from patients with metastasis and its non-tumor adjacent regions (N-TAR). Messenger RNA (mRNA) levels were determined by quantitative Polymerase Chain Reaction (qPCR) and adjusted by Actin Beta (ACTB) expression levels. (**d**-**e**) Comparison of GPR107 expression levels between normal prostate (*n*), primary prostate tumor (P) and metastatic Castration Resistant Prostate Cancer (mCRPC) samples obtained from two in silico databases (Grasso/Varambally; (**d**) and (**e**), respectively). Asterisks (* *p* < 0.05; ** *p* < 0.01; *** *p* < 0.001; **** *p* < 0.0001) indicate statistically significant differences between groups. AUC: Area Under the Curve.

We also analyzed the expression of GPR107 in an independent cohort of more aggressive PCa (*n* = 67; Gleason score 7–10; Table 2). We found that GPR107 expression was significantly higher in primary PCa samples from patients with mHSPC compared to those without metastasis (Figure 2a). Indeed, ROC analysis indicated that GPR107 expression significantly discriminated between metastatic vs. non-metastatic patients (*p* = 0.0064; Figure 2b). These observations were also corroborated at the protein level by GPR107 IHC, which clearly indicated that GPR107 staining was negligible in benign prostate gland epithelium (N-TAR; Figure 2c), while it was highly intense in the metastatic regions (Figure 2c). Remarkably, GPR107 overexpression was also corroborated in metastasis from metastatic CRPC (mCRPC) compared to primary prostate tumors using two independent external in silico cohorts of patients obtained from different databases available online (Grasso (Figure 2d) and Varambally (Figure 2e)), while this overexpression was not found in mHSPC samples obtained from Lapointe, Taylor and Tomlins in silico cohorts [26,27,28] (Figure S3).

### 3.3. Silencing of GPR107 Reduces Aggressiveness Parameters in Androgen-Independent PCa Cells

**Figure 3:**
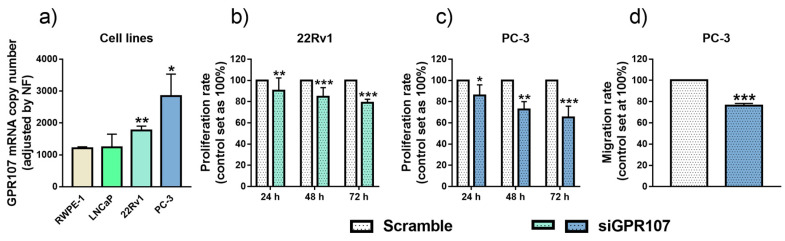
Screening of G protein-coupled receptor 107 (GPR107) expression level and functional effects of its silencing in normal and tumor prostate cell lines. (**a**) Comparison of GPR107 expression levels between a non-tumor prostate cell line (RWPE-1) and different Prostate Cancer (PCa) cell lines (LNCaP, 22Rv1 and PC-3). Messenger RNA (mRNA) levels were determined by quantitative Polymerase Chain Reaction (qPCR) and adjusted by a normalization factor (NF) generated by the combination of Actin Beta (ACTB) and Glyceraldehyde-3-Phosphate Dehydrogenase (GAPDH) expression levels. (**b)**–(**c**) Proliferation rate of 22Rv1 (**b**) and PC-3 (**c**) cells after 24, 48 and 72 h of GPR107-silencing. (**d**) Migration rate of PC-3 cells after 24 h of GPR107-silencing. In (**b**)–(**d**), data were represented as percent of scrambled cells (set at 100%). Asterisks (* *p* < 0.05; ** *p* < 0.01; *** *p* < 0.001) indicate statistically significant differences between groups. siGPR107: small interferent RNA against GPR107.

To examine the possible functional role of GPR107 on PCa cell malignant features, we initially examined its expression levels on different prostate cell lines (normal (RPWE-1) and PCa (androgen-dependent LNCaP, as well as androgen-independent 22Rv1 and PC-3 cells)). Specifically, GPR107 expression was significantly higher in androgen-independent, 22Rv1 and PC-3, cells compared to the LNCaP and the normal RWPE-1 cells (Figure 3a). Based on these results, 22Rv1 and PC-3 were selected as suitable models to analyze the functional consequences of GPR107 silencing. Interestingly, silencing of GPR107, confirmed by qPCR and western-blot (Figure S1), clearly decreased cell proliferation in both 22Rv1 (Figure 3b) and PC-3 (Figure 3c) cells (at 24, 48 and 72 h vs. scramble-transfected control). Moreover, GPR107 silencing markedly decreased migration rate in PC-3 cells (Figure 3d).

### 3.4. Silencing of GPR107 Modulates the Expression of Key Genes and Oncogenic Signaling Pathway in Androgen-Independent PCa Cells

**Figure 4:**
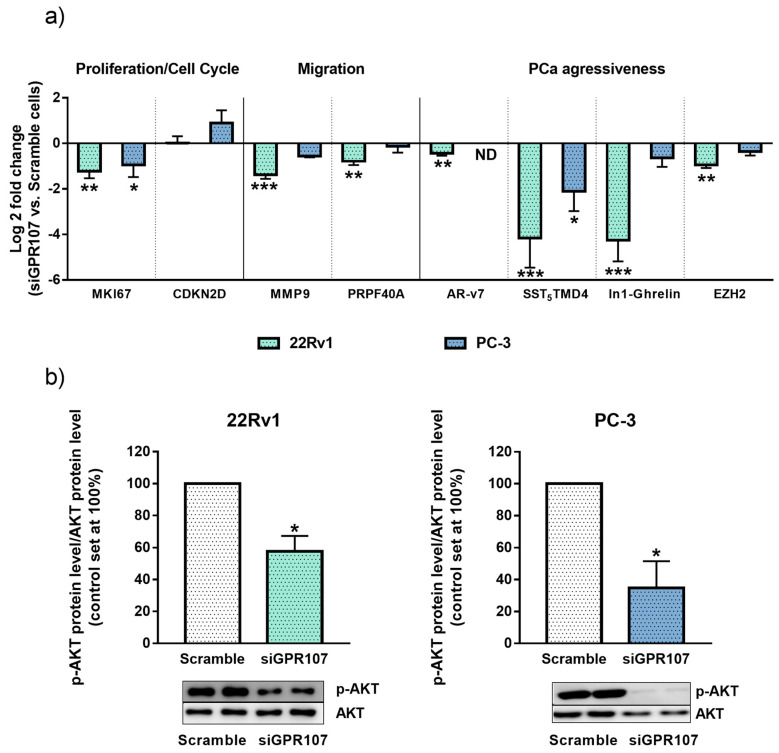
Molecular consequences of G protein-coupled receptor 107 (GPR107)-silencing in androgen-independent Prostate cancer (PCa) cells. (**a**) Fold change of markers of proliferation (Marked of Proliferation Ki-67 (MKI67)), cell cycle inhibition (Cyclin Dependent Kinase Inhibitor 2D (CDKN2D)), migration (Matrix Metallopeptidase 9 and Pre-MRNA Processing Factor 40 Homolog A (MMP9 and PRPF40A, respectively)) and aggressiveness (Androgen Receptor variant 7 (AR-v7), Somatostatin Receptor 5 Transmembrane Domain 4 variant (SST_5_TMD4), Intron 1-retained ghrelin variant (In1-Ghrelin) and Enhancer Of Zeste Homolog 2 (EZH2)) in androgen-independent cells (22Rv1 and PC-3) in response to GPR107-silencing compared to scrambled cells. Messenger RNA (mRNA) levels were determined by quantitative Polymerase Chain Reaction (qPCR), adjusted by Actin Beta (ACTB) expression levels and represented as log 2 of fold change of expression levels small interferent RNA(siRNA)-treated/scrambled-cells). (**b)** Protein levels of phospho-AKT (p-AKT) in response of GPR107-silencing in androgen-independent PCa cells (22Rv1 and PC-3). p-AKT levels were normalized by total AKT protein levels. Protein data were represented as percent of scrambled cells (set at 100%). Asterisks (* *p* <0.05; ** *p* < 0.01 and *** *p* < 0.001) indicate statistically significant differences between GPR107-silencing and scrambled cells. siGPR107: small interferent RNA against GPR107.

To identify the molecular consequences of GPR107 silencing in androgen-independent PCa cells, we analyzed the expression levels of key genes related to proliferation/cell-cycle, migration and aggressiveness. Specifically, we found that the silencing of GPR107 in 22Rv1 cells significantly decreased the expression levels of the proliferation marker Ki-67 (MKI67), of genes involved in migration process (Matrix Metallopeptidase 9 and Pre-MRNA Processing Factor 40 Homolog A (MMP9 and PRPF40A, respectively)) as well as of key genes associated to PCa aggressiveness such as the oncogenic splicing variants AR-v7, SST_5_TMD4, Intron 1-retained ghrelin splicing variant (In1-ghrelin) and the Enhancer Of Zeste Homolog 2 (EZH2) (Figure 4a). In PC-3 cells, a significant decrease in the expression levels of MKI67 and SST_5_TMD4 and a trend for a significant decrease of MMP9, In1-ghrelin and EZH2 and for a significant elevation in the expression of cell-cycle suppressor Cyclin Dependent Kinase Inhibitor 2D (CDKN2D) was also found in response to GPR107 silencing (Figure 4a).

Moreover, we sought to identify the downstream consequences of GPR107 silencing by analyzing the AKT route, a key signaling pathway in PCa cells. Specifically, phosphorylation levels of AKT were down-regulated in response to GPR107 silencing in both 22Rv1 and PC-3 cells (Figure 4b). We also analyzed the modulation of ERK pathway in response to GPR107 silencing but this signaling pathway was not significantly altered by this experimental intervention.

### 3.5. NST Treatment Exerts Antitumor Effects in Androgen-Independent PCa Cells

**Figure 5:**
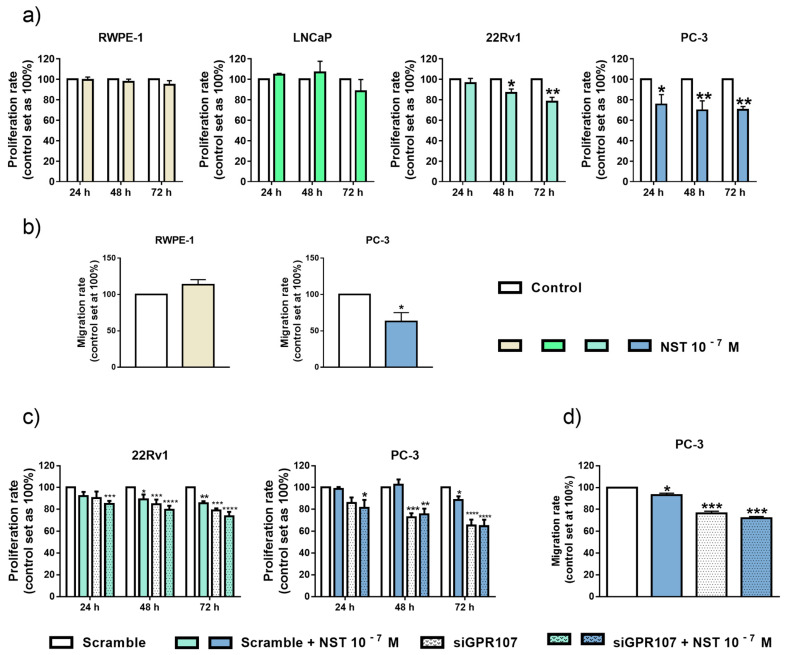
Functional effects after neuronostatin (NST) treatment and after the combination of NST and G protein-coupled receptor 107 (GPR107)-silencing treatment in prostate cell lines. (**a**) Proliferation rate of normal prostate (RWPE-1) and Prostate Cancer (PCa) (LNCaP, 22Rv1 and PC-3) cells in response to NST treatment (10^–7^ M; after 24, 48 and 72 h). (**b**) Migration rate of normal prostate (RWPE-1) and PCa (PC-3) cells after 24 h of NST treatment (10^–7^ M). Proliferation (**c**) and migration (**d**) rate of androgen-independent PCa cells in response to NST and GPR107-silencing alone or in combination. Data were represented as percent of vehicle-treated cells (set at 100%). Asterisks (* *p* < 0.05; ** *p* < 0.01 and *** *p* < 0.001) indicate statistically significant differences between groups. siGPR107: small interferent RNA against GPR107.

We next tested the direct effects of NST on proliferation and migration of normal and PCa cells (Figure 5). Incubation with NST did not alter proliferation or migration rate in normal (RWPE-1) cells (Figure 5a,b, left panels). Likewise, NST treatment did not alter proliferation of androgen-dependent LNCaP cells (Figure 5a). However, similar to that previously found with GPR107 silencing (Figure 3), NST treatment significantly decreased proliferation rate of androgen-independent 22Rv1 and PC-3 cells (Figure 5a), as well as the migration of PC-3 cells (Figure 5b).

### 3.6. Actions of NST Treatment and GPR107 Silencing are Similar, and Functionally Connected, in Androgen-Independent PCa Cells

In order to analyze whether the actions of NST in the proliferation and/or migration rates of androgen-independent PCa cells could be functionally associated to GPR107, we next tested the direct effects of NST alone or in combination with GPR107 silencing. As observed previously, NST treatment or GPR107 silencing alone inhibited the proliferation of 22Rv1 and PC-3 (Figure 5c), as well as the migration of PC-3 cells (Figure 5d). These inhibitory actions of NST treatment or GPR107 silencing were virtually similar in 22Rv1 cells, while the actions of GPR107 silencing seem to be higher compared to NST treatment in PC-3 cells (Figure 5c,d). Furthermore, the combination of NST treatment and GPR107 silencing did not exert higher, additive or synergistic, effects compared with both experimental conditions alone (Figure 5c,d), which might suggest that the inhibitory actions evoked by NST treatment and GPR107 silencing could be mediated through similar mechanisms and/or signaling pathways.

**Figure 6:**
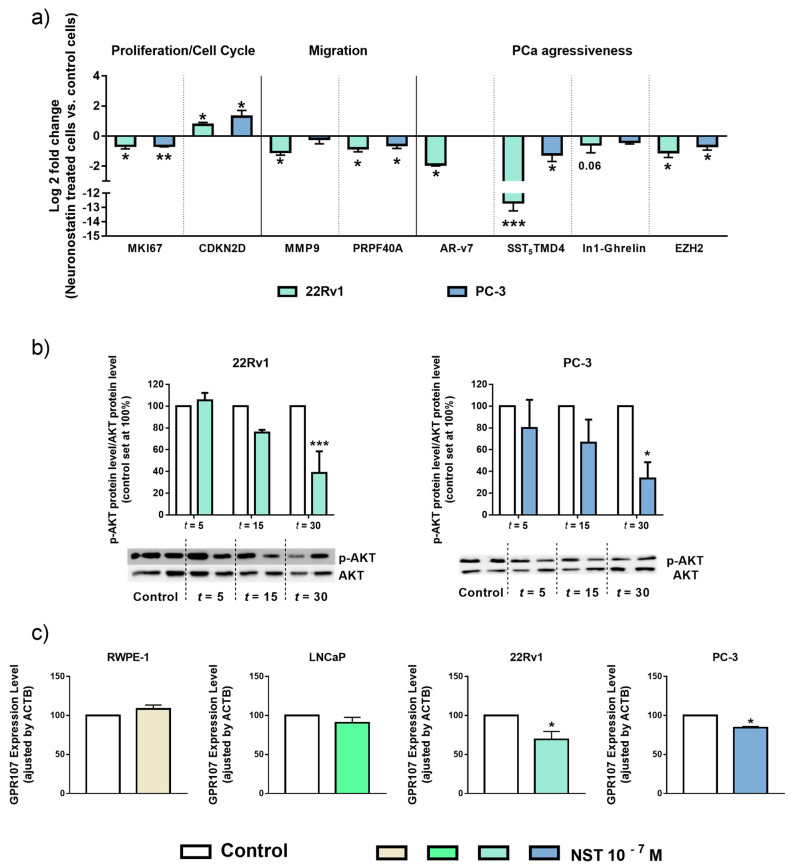
Molecular consequences of neuronostatin (NST) treatment in prostate cell lines. (**a**) Fold change of markers of proliferation (Marked of Proliferation Ki-67 (MKI67)), cell cycle inhibition (Cyclin Dependent Kinase Inhibitor 2D (CDKN2D)), migration (Matrix Metallopeptidase 9 and Pre-MRNA Processing Factor 40 Homolog A (MMP9 and PRPF40A, respectively)) and aggressiveness (Androgen Receptor variant 7 (AR-v7), Somatostatin Receptor 5 Transmembrane Domain 4 variant (SST_5_TMD4), Intron 1-retained ghrelin variant (In1-Ghrelin) and Enhancer Of Zeste Homolog 2 (EZH2)) in androgen-independent Prostate Cancer (PCa) cells (22Rv1 and PC-3) after NST treatment (10^−7^ M) compared to vehicle-treated cells. Messenger RNA (mRNA) levels were determined by quantitative Polymerase Chain Reaction (qPCR), adjusted by Actin Beta (ACTB) expression levels and represented as log2 of fold change of expression levels (NST-treated/vehicle-treated cells). (**b**) Protein levels of phospho-AKT in response of NST treatment in androgen-independent PCa cells (22Rv1 and PC-3) after 5 (*t* = 5), 15 (*t* = 15) and 30 (*t* = 30) minutes of exposition. Phospho-AKT (p-AKT) levels were normalized by total AKT protein levels. Protein data were represented as percent of vehicle-treated cells (set at 100%). (**c**) GPR107 expression levels after 24 h of NST treatment in prostate cell lines. mRNA levels of GPR107 were determined by qPCR and adjusted by ACTB expression levels. Asterisks (* *p* < 0.05; ** *p* < 0.01 and *** *p* < 0.001) indicate statistically significant differences between NST treatment and vehicle-treated cells.

Supporting this notion, we found that treatment with NST induced a molecular response virtually similar to that previously observed with GPR107 silencing in term of the modulation of the expression levels of key genes related to proliferation/cell cycle, migration and aggressiveness (MKI67, CDKN2D, MMP9, PRPF40A, AR-v7, SST_5_TMD4, In1-ghrelin and EZH2; Figure 6a), as well as inhibition of AKT (Figure 6b), but not ERK, signaling pathway.

Finally, we found that NST administration did not alter the expression levels of GPR107 in normal RWPE-1 cells or in androgen-dependent LNCaP cells, whereas it significantly decreased GPR107 levels in androgen-independent 22Rv1 and PC-3 cells (Figure 6c).

## 4. Discussion

PCa is the most prevalent form of cancer and the second cause of death in men worldwide [1,5]. The management of PCa has improved in recent years with the use of novel imaging and treatment procedures; however, locally advanced or metastatic PCa still has the potential to develop often into a lethal phase as no curative paradigm yet exists. Thus, new molecular avenues are urgently needed to better diagnose, predict their prognosis and tumor behavior and to provide tools to develop better therapeutic tools that prolong patient survival. In line with this, the somatostatin-SSTRs system represents a useful source of therapeutic targets and tools to treat various endocrine-related tumors, owing to its pleiotropic role encompassing from whole body homeostasis to cancer cell functioning in different tumor types, where this system commonly acts to inhibit multiple processes, such as hormone secretion and cell proliferation, migration and invasion [10,13,32,40,41]. Notwithstanding, earlier, limited studies using somatostatin-analogues (SSAs) found no benefits in overall survival in PCa patients [14,42]. More recently, we reported that one of the mechanistic reasons of this clinical failure might be the presence and relevant oncogenic role of the spliced SST_5_TMD4 variant in PCa cells [11]. In this scenario, the recently discovered functional system associated to the somatostatin regulatory axis comprised by NST and GPR107 has been shown to exert diverse physiologic activities at the central and peripheral level [15,19,43]; however, their presence and possible functional role in the pathophysiology of PCa is still unknown.

Accordingly, we initially explored this issue by testing for the first time the GPR107 presence and functional relevance in PCa, using diverse experimental and analytical approaches. This revealed that GPR107 is present in a high proportion of PCa samples and is overexpressed, at both mRNA and protein levels, in PCa tissues, as compared to non-tumor tissues in two independent cohorts of human samples. Moreover, elevated expression of GPR107 was found in primary tissues from patients diagnosed with localized PCa and in patients with more aggressive, metastatic PCa. Most importantly, GPR107 overexpression was evidenced in samples from patients with mHSPC compared to those without metastasis. In this line, although we acknowledge that a limitation of our study is the lack of analyzed mCRPC samples, the results presented herein compare favorably with data from two independents external in silico cohorts of patients with mCRPC (Grasso and Varambally datasets). As a result, ROC analysis revealed that GPR107 expression could discriminate between patients that developed metastasis vs. those that did not. Even more important is the fact that GPR107 expression levels were directly associated with other relevant clinical parameters of PCa-aggressiveness (i.e., tumor stage and vascular invasion and presence of metastasis) as well as with the expression levels of key molecular markers of PCa-aggressiveness (e.g., CDK2, VEGFR, IL6R) [44,45,46]. These results reinforce the notion of a causal link between dysregulation of GPR107 expression and PCa aggressiveness, suggesting that this receptor may play a significant pathophysiological role in PCa cells. The contention of the potential oncogenic role of GPR107 in PCa is in line with a previous report indicating that GPR107 drives self-renewal and tumorigenesis of liver tumor initiating cells [47]. Thus, our results offer original evidence to suggest that GPR107 dysregulation may play a relevant functional pathophysiological role in PCa and could provide new tools as a diagnostic and prognostic biomarker and/or therapeutic target for PCa, especially for metastatic PCa, given its association with clinical and molecular features of aggressiveness.

These initial results led us to further explore the functional pathophysiological role of GPR107 in PCa cell models. The first approach was to assess the effect of GPR107-silencing on cell proliferation and migration, two parameters tightly linked to tumor growth and metastasis, some of the main clinical problems associated to PCa. Silencing of GPR107 decreased proliferation and migration in two representative models of CRPC pathology (i.e., 22Rv1 and PC-3 cells), demonstrating that GPR107 is functionally active in AI-PCa cells and that its presence is directly associated with their aggressiveness features. These results are in agreement with a previous evidence indicating that GPR107 expression knock-down decreased aggressiveness features in liver tumor initiating cells (i.e., impaired tumor initiation, self-renewal and invasion capacities) [47]. Additionally, the functional data observed in the present study in response to GPR107-silencing (i.e., decreased proliferation and migration capacity) could suggest that GPR107 bears a constitutive functional activity in PCa cells. Remarkably, this is neither the sole nor the first time that a constitutive activation of receptor belonging to the somatostatin-related regulatory system has been reported since various SSTRs have been demonstrated to display a relevant degree of ligand-independent constitutive activity in different cell systems [48]. The mechanisms underlying the effect of GPR107 are yet unknown, and future studies should ascertain whether they are mediated by ligand-dependent or -independent (e.g., receptor constitutive activity) actions. Nonetheless, these observations unveiled new conceptual and functional avenues in PCa, with potential therapeutic implications, which warrant further investigation.

To interrogate the signaling pathways and molecular elements mediating GPR107 actions in PCa cells, we used AI-PCa cells (22Rv1 and PC-3 cells) as model. This revealed that GPR107 might exert its tumor-associated functions through modulation of several molecular/signaling pathways, including a decreased activation (basal phosphorylation) of AKT signaling pathway, which has been shown to be a key oncogenic-signaling pathway and cooperate in different tumor pathologies, including PCa, to promote malignancy, drug resistance and CRPC development [49,50]. In fact, this result indicating that silencing of GPR107 may inhibit cell proliferation/migration via negative regulation of AKT pathway is a common mechanism that has been previously reported with other components of the somatostatin system in different tumor types, including PCa [10,41,51,52,53]. Moreover, silencing of GPR107 in AI-PCa cells decreased the expression levels of MKI67, a well-known proliferation marker associated to biochemical recurrence in PCa [54], and, in PC-3 cells, tended to increase the expression of CDKN2D, a cell-cycle inhibitor involved in the growth arrest at senescence of PCa cells [55]. Similarly, GPR107-silencing resulted in a decrease of MMP9 and PRPF40A, genes involved in the process of migration and cytoskeletal regulation, respectively [56,57]. Interestingly, we also found that the decrease in the aggressiveness of PCa cells in response to GPR107-silencing could also involve a diminished expression of the splicing variants SST_5_TMD4, In1-ghrelin and AR-v7, as well as EZH2, four elements previously reported as key oncogenic factors in PCa and/or main drivers of CRPC [11,30,58,59]. Interestingly, GPR107 expression was correlated with SST_5_TMD4 but not with SST_5_, In1-ghrelin or AR-v7 expression in the more aggressive cohort of PCa samples (Cohort 2, Figure S2), which reinforces the idea of a role for GPR107 as potential therapeutic target in PCa, in that we have recently reported that SST_5_TMD4 is a key pathophysiological component in this cancer type [11,53]. This is consistent, actually, with the fact that GPR107 silencing in AI-PCa cells induced significant changes in key factors involved in and associated with SST_5_TMD4-related pathways, such as modulation of AKT-signaling pathway and MKI67 expression [11,53]. Moreover, these results have special relevance because GPR107 silencing was able to consistently decrease SST_5_TMD4 expression in all the AI-PCa models tested herein, in which we previously demonstrated that overexpression of SST_5_TMD4 is directly associated to the inefficiency of SSA therapy (i.e., octreotide treatment) in PCa cells and other tumor types [11,32,60,61,62] as well as of other drugs currently used for the treatment of PCa (i.e., abiraterone or enzalutamide) [11].

Finally, in order to further explore the potential utility of the NST-GPR107 system as therapeutic target, functional and mechanistic studies were performed in response to NST treatment in PCa cells [16,17,19]. Our results revealed for the first time that NST treatment evoked virtually similar antitumor effects (i.e., reduction of proliferation and migration capacity) to those previously observed with GPR107-silencing in AI-PCa cells. Similarly, treatment with NST induced a signaling and molecular regulatory response comparable to that of the GPR107-silencing treatment (i.e., inhibition of AKT signaling pathway and modulation of the expression of MKI67, CDKN2D, MMP9, PRPF40A, AR-v7, SST_5_TMD4, In1-ghrelin, AR-v7 and EZH2), which reinforces the idea that the antitumor actions observed in response to GPR107-silencing or NST treatment might be functionally connected and mediated through similar mechanisms and/or signaling pathways. Moreover, in support of this notion is the fact that the combined treatment of NST and GPR107-silencing did not modify the anti-proliferative/migratory actions of both treatments individually in AI-PCa cells. Furthermore, we also found that NST administration significantly decreased GPR107 levels in AI-PCa cells, which might indicate that the antitumor actions of NST might be exerted, at least in part, by decreasing the expression levels of GPR107 in AI-PCa cells. Therefore, although further studies would be necessary before a precise and unequivocal conclusion can be reached in this regard, all these in vitro experiments suggest that GPR107 may exert its oncogenic role through the induction of a constitutive activation of AKT signaling pathway, which may lead to changes in the expression of prostate cancer-related genes (i.e., the overexpression of MKI67, MMP9, PRPF40A, SST5TMD4, AR-v7, In1-ghrelin, EZH2 and the reduction of CDKN2D gene expression) and that NST may be exerting its anti-tumor actions by decreasing GPR107 expression, at least in AI-PCa cells.

## 5. Conclusions

Taken together, our results provide the first identification of the presence and functional role of the NST-GPR107 system in PCa, which enabled to demonstrate a relevant pathological function and therapeutic potential of this regulatory system in PCa cells in vitro and in vivo. Indeed, our results demonstrate that GPR107 is overexpressed in PCa, especially in metastatic-PCa, and its expression levels are associated to key aggressiveness features of PCa suggesting that this receptor could represent a valuable diagnostic tool and a promising prognostic biomarker in PCa patients. Moreover, we have demonstrated that both GPR107-silencing and NST treatment altered key pathophysiological parameters in PCa in vitro, including a reduction of cell proliferation and migration and modulation of the expression levels of relevant molecular markers (e.g., MKI67, SST_5_TMD4, AR-v7, etc.), possibly through the modulation of the key AKT pathway. Altogether, the translational research implications of these findings indicate that GPR107 has a functional role in the pathophysiology of PCa and invites to suggest that pharmacological treatments specifically targeting this receptor, including NST treatment, could become a promising option to treat patients with PCa, specially metastatic PCa, providing a relevant clinical conclusion, which should be soon tested for their use in humans.

## Figures and Tables

**Table 1:** Demographic, biochemical and clinical parameters of the patients who underwent radical prostatectomies (Cohort 1).

Parameter	
Patients (*n*)	84
Age, years (median (IQR))	61 (57–66)
PSA levels, ng/mL (median (IQR))	5.2 (4.2–8.0)
GS (*n*; %)	GS 6 (8; 9.52%), GS 7 (73; 86.90), GS 8 (3; 3.57%)
SigPCa (*n* (%))	76 (90.5%)
pT ≥ 3a (*n* (%))	59 (70.2%)
PI (*n* (%))	72 (85.7%)
VI (*n* (%))	8 (9.52%)
Recurrence (*n* (%))	35 (41.7%)
Metastasis (*n* (%))	0 (0%)

PSA: Prostate specific antigen; GS: Gleason Score; SigPCa: Significant prostate cancer, defined as Gleason score ≥ 7; pT: Pathological primary tumor staging; PI: Perineural invasion; VI: Vascular invasion.

**Table 2:** Demographic, biochemical and clinical parameters of the patients who underwent prostate biopsy (Cohort 2).

Parameter	
Patients (*n*)	67
Age, years (median (IQR))	75 (69–81)
PSA levels, ng/mL (median (IQR))	62.0 (36.2–254.5)
GS (*n*; %)	GS 7 (18; 26.86%), GS 8 (20; 29.85%)
	GS 9 (24; 35.82%), GS 10 (5; 7.46%)
SigPCa (*n* (%))	67 (100%)
Metastasis (*n* (%))	27 (40.3%)

PSA: Prostate specific antigen; GS: Gleason Score; SigPCa: Significant prostate cancer, defined as Gleason score ≥ 7.

## Supplementary Materials

The following are available online at https://www.mdpi.com/2077-0383/9/6/1703/s1. Figure S1: Validation of GPR107-silencing in androgen-independent PCa cell line 22Rv1 after 48h of GPR107 siRNA transfection. Figure S2: Correlation between GPR107 expression levels and different key oncogenic factors in primary PCa samples from patients with metastasis. Figure S3. Comparison of GPR107 expression levels between non-Tumoral (N), primary tumor (PT), mHSPC specimens (M) obtained from three in silico databases (Lapointe, Taylor and Tomlins). Table S1: Specific primers for human transcripts used in this study.
