# Supplementary material for: Unleashing the Diagnostic, Prognostic and Therapeutic Potential of the Neuronostatin/GPR107 System in Prostate Cancer
Source: J Clin Med. 2020 Jun 2;9(6):1703. doi: 10.3390/jcm9061703 (PMC7355908; doi:10.3390/jcm9061703)
Supplement: Supplementary file 1 [file jcm-09-01703-s001.pdf]

Supplementary material

Table S1. Specific primers for human transcripts used in this study.

Gene	Accession Number	Primer Sequence (Sense)	Primer Sequence (Antisense)	Product Size (bp)
MKI67	NM_002417	GACATCCGTATCCAGCTTCCT	GCCGTACAGGCTCATCAATAAC	139
CDKN2D	NM_001800.4	AACCGCTTCGGCAAGAC	GCTGGCACCTTGCTTCA	67
MMP9	NM_004994.2	CAGTGCCATGTAAATCCCCA	CACCTCCACTCCTCCCTTTC	102
PRPF40A	NM_017892.3	GCTCGGAAGATGAAACGAAA	TGTCCTCAAATGCTGGCTCT	130
AR-v7	NM_001348061.1	CAGGGATGACTCTGGGAAAA	TGAGGCAAGTCAGCCTTTCT	87
SSTR5	NM001053	CTGGTGTTTGCGGGATGTT	GAAGCTCTGGCGGAAGTTGT	183
SST$_5$*TMD4*	NM_001172560.1	TACCTGCAACCGTCTGCC	AGCCTGGGCCTTTCTCCT	98
In1-Ghrelin	GU942497.1	TCTGGGCTTCAGTCTTCTCC	GTTCATCCTCTGCCCCTTCT	215
EZH2	NM_004456.5	CACTCCTTTCATACGCTTTTCTG	TGTTTCTGTGTTCTTCCGCTTA	119
CDK2	NM_001798.4	GCTCTCACTGGCATTCCTCTT	GAGGTTTAAGGTCTCGGTGG	109
CDK4	NM_000075.3	ACAGTTCGTGAGGTGGCTTT	TACCTTGATCTCCCGGTCAG	111
MMP3	NM_002422.5	ACTAGCAAGGACCTCGTTTTCA	AGGGTGTGGATGCCTCTTG	79
IL6-R	NM_000565.4	AAGGCTGTGCTCTTGGTGAG	TGGGACTCCTGGGAATACTG	83
VEGFR	NM_002253.3	TCTCTGCCTACCTCACCTGTTT	TGACTGATTCCTGCTGTGTTG	89
GPR107	NM_001136557.2	AACCAGACCCAGAAGACACAA	GACACTGCCCCACCATTATT	109
ACTB	NM_001101	ACTCTTCCAGCCTTCCTTCCT	CAGTGATCTCCTTCTGCATCCT	176
GADPH	NM_002046	AATCCCATCACCATCTTCCA	AAATGAGCCCCAGCCTTC	122

NCBI accession number, primers sequences and expected product sizes are included.

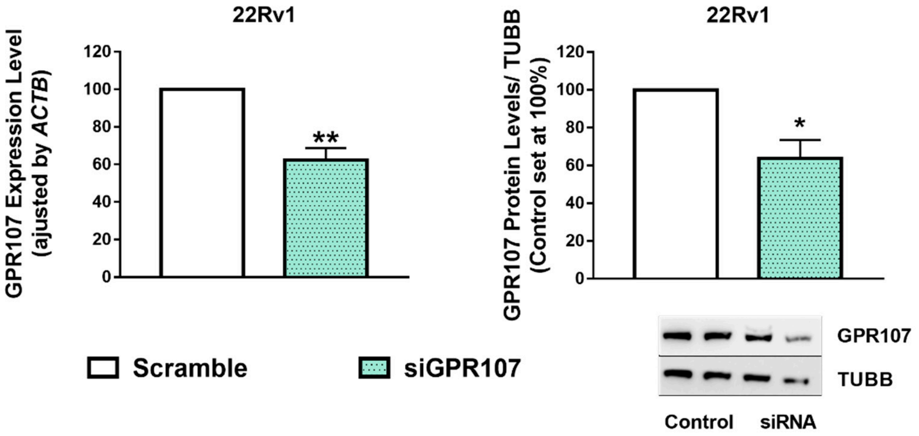

Figure S1. Validation (at mRNA and protein levels by qPCR and Western blot) of GPR107-silencing in androgen-independent PCa cell line 22Rv1 after 48 h of GPR107 siRNA transfection. mRNA levels of *GPR107* were determined by qPCR and adjusted by *ACTB* expression levels. GPR107 protein level was normalized by Β-Tubulin (TUBB) protein levels. Data were represented as percent of scrambled cells (set at 100%). Asterisks (* p <0.05; ** p <0.01) indicate statistically significant differences between GPR107-silencing and scrambled cells.

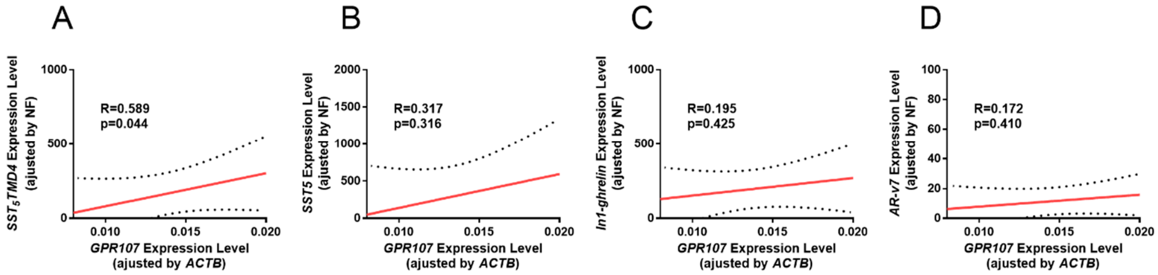

Figure S2. Correlation between *GPR107* expression levels and different key oncogenic factors in primary PCa samples from patients with metastasis. Correlation of *GPR107* expression level and *SST_5TMD4* (A), *SST_5* (B), *In1-Ghrelin* (C) and *AR-v7* (D) expression in primary PCa samples from patients with metastasis (cohort 2). mRNA levels were determined by qPCR and adjusted by *ACTB* expression levels or by normalization factor (NF) generated by the combination of *ACTB* and *GAPDH* expression levels.

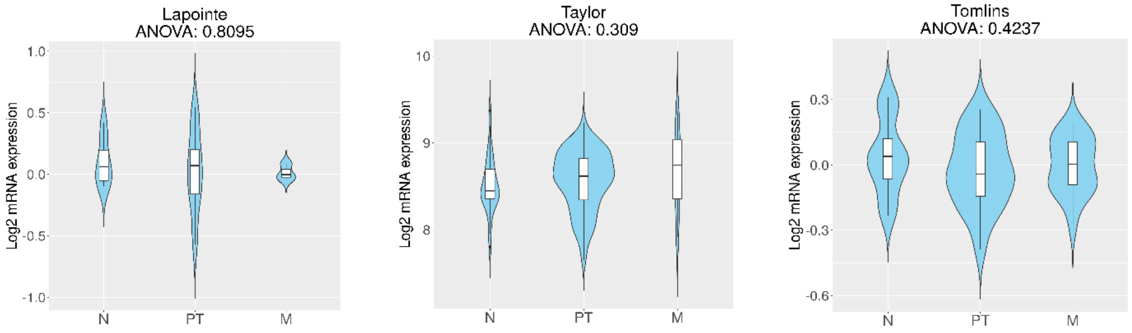

Figure S3. Comparison of GPR107 expression levels between non-Tumoral (N), primary tumor (PT), mHSPC specimens (M) obtained from three in silico databases (Lapointe, Taylor and Tomlins). The Y-axis represents the Log2-normalized gene expression. An ANOVA test is performed in order to compare the mean gene expression among groups.
